# Genetic diversity and conservation of a rare *Miconia* species (Melastomataceae) from the Brazilian Atlantic Forest

**DOI:** 10.1007/s10709-026-00296-1

**Published:** 2026-10-01

**Authors:** Gabriela Goebel, Ana Flávia Augustin, Eduardo Koerich Nery, Yohan Fritsche, Valdir Marcos Stefenon, Duane Fernandes Lima, Mayara Krasinski Caddah

**Affiliations:** 1 Programa de Pós-graduação em Biologia de Fungos, Algas e Plantas, Centro de Ciências Biológicas, Departamento de Botânica, Universidade Federal de Santa Catarina, R. Eng. Agronômico Andrei Cristian Ferreira, 216, Florianópolis, SC 88040-535 Brasil; 2 Programa de Pós-graduação em Recursos Genéticos Vegetais, Centro de Ciências Agrárias, Departamento de Fitotecnia, Universidade Federal de Santa Catarina, Rod. Admar Gonzaga, 1346, Florianópolis, SC 88034-000 Brasil; 3 Centro de Ciências Naturais e Humanas, Universidade Federal do ABC, Alameda da Universidade, São Bernardo do Campo, SP 09606-045 Brasil; 4 Setor de Ciências Agrárias e Ambientais, Departamento de Ciências Biológicas, Universidade Estadual do Centro-Oeste, Alameda Élio Dalla Vecchia, 838, Guarapuava, PR 85040-167 Brasil

**Keywords:** Conservation genetics, Microsatellites, Populations genetics, Rare species, SSRs

## Abstract

Biodiversity is undergoing an unprecedented global decline, and genetic diversity plays a fundamental role in ensuring species persistence. *Miconia ulei*, an endemic species from the eastern Atlantic Forest in Santa Catarina, southern Brazil, is rare and has few known populations. We investigated genetic diversity and population structure using microsatellite markers and assessed its conservation status following IUCN guidelines and by incorporating genetic parameters in the evaluation. Five extant populations were sampled across the species’ known distribution, and twelve species-specific microsatellite markers were developed. Populations exhibited low to moderate levels of genetic diversity. Genetic differentiation among populations was generally low, although one population showed a distinct genetic structure. No isolation by distance was detected, suggesting that factors other than geographic distance may influence patterns of gene flow. Evidence of historical reductions in population size was detected in most populations. Together, these findings indicate that *M. ulei* has limited genetic variation and small, fragmented populations, increasing its vulnerability to future genetic loss. Our results support the need for conservation actions, including the formal inclusion of *M. ulei* in official Red Lists and the maintenance of habitat connectivity to promote long-term population persistence. As the first population genetic assessment of *M. ulei*, this study provides an important baseline for conservation planning and highlights the need for conservation genetic studies of other rare Atlantic Forest species.

**Supplementary Information:**

The online version contains supplementary material available at https://doi.org/10.1007/s10709-026-00296-1.

## Introduction

Biodiversity is experiencing a global decline at unprecedented rates, and genetic diversity is a key component for its maintenance worldwide (Hoban et al. 2020; IPBES 2019). Anthropic impacts, such as habitat loss and invasive species, are promoting reductions in genetic diversity, limiting the resilience of species to current climate change (Young et al. 1996; Frankham 2019a; Waldvogel et al. 2020). In this context, The Kunming–Montreal Global Biodiversity Framework (GBF), which establishes conservation targets for 2030 and 2050 (CBD 2022), prioritizes the maintenance of genetic diversity in both wild and domesticated species. Hence, genetic data have become an essential tool for monitoring populations and species and for guiding conservation actions (Hvilsom et al. 2022; Liddel et al. 2021; Ralls et al. 2018).

Genetic parameters can inform key aspects of populations that are under extinction risk (Gargiulo et al. 2025). In this context, increasing efforts have been made to incorporate genetic data into extinction risk assessments, including those conducted by the International Union for Conservation of Nature (IUCN), a global authority on assessing species extinction risk and guiding conservation strategies (Garner et al. 2020; McLaughlin et al. 2025). In particular, three genetic parameters are essential for conservation: population genetic diversity, population genetic structure, and effective population size (Hoban et al. 2020; McLaughlin et al. 2025). Population genetic diversity is usually measured by number of alleles and levels of heterozygosity, with lower values of both generally indicating lower adaptive potential in changing environments (Hartl and Clarck 2010). Population genetic structure reveals how alleles are spatially distributed, with greater values indicating enhanced endogamy and possible gene flow barriers (Eriksson et al. 2013). In turn, the effective population size represents the number of individuals contributing genetically to future generations, with smaller sizes indicating greater susceptibility to extinction (Hoban et al. 2020; Frankham et al. 2014). Together, these genetic parameters can identify populations with greater extinction risk, guiding conservation efforts.

The Atlantic Forest (AF) is a global conservation hotspot in Eastern Brazil (Myers 2000), holding a great percentage of endemic species under high extinction risk (Mittermeier et al. 2004; Vancine et al. 2024). The AF is a phytogeographic domain that harbors distinct vegetation types, such as mangroves, coastal woodlands (*restingas*), lowland ombrophilous forests, montane mixed forests, and semi-decidous forests. Post-colonial deforestation has reduced the AF to 12% of its original area, now mostly fragmented in small secondary remnants (SOS Mata Atlântica 2024). These reduction and fragmentation processes have reduced plant population sizes and restricted gene flow for plant species in the AF (Melo and Franceschinelli 2016; Pereira et al. 2022; Barreto et al. 2023). For instance, in the species *Dalbergia nigra* (Fabaceae), the genetic diversity found in different forest remnants was lower than expected, with high differentiation between populations, suggesting isolation and low gene flow (Barreto et al. 2023). Similar patterns have been predicted for fragmented plant populations in general (Aguilar et al. 2008). Small and reproductively isolated populations are expected to become more susceptible to genetic drift, which can drastically reduce genetic diversity and increase extinction risk (Kageyama and Gandara 1988; Young et al. 1996; Frankham et al. 2002; Ralls et al. 2018; Frankham 2019a). Despite theoretical expectations, the impacts of area reduction and fragmentation have been primarily documented for common plant species in the AF (Forti et al. 2014; Carvalho et al. 2020), whereas rare species remain poorly understood (e.g. Augustin et al. 2025).

**Fig. 1:**
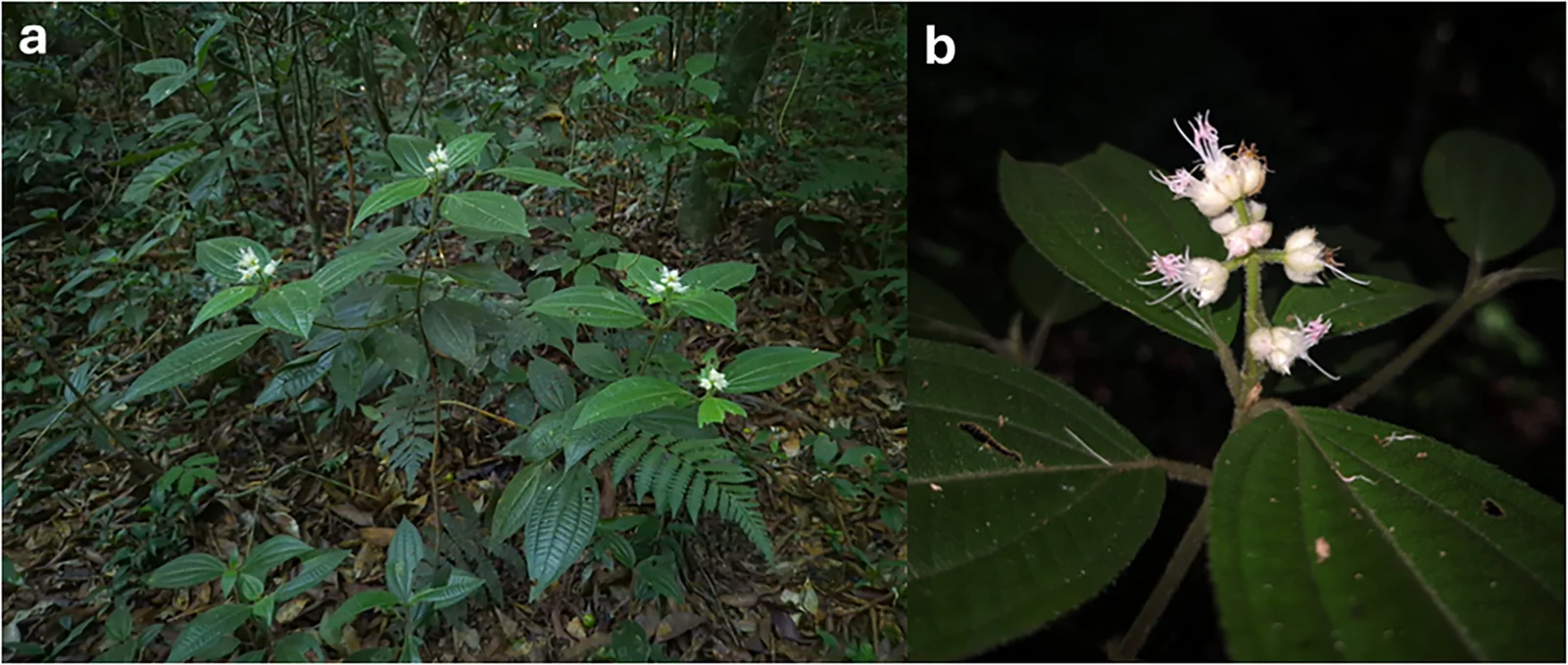
**a** Individual of *Miconia ulei* in its natural habitat in Southern Atlantic Forest. **b** Detail of the inflorescence of *M. ulei*, showing persistent bracteoles

*Miconia ulei* (Cogn.) R.Goldenb. is an endemic species from the Southern AF, restricted to the state of Santa Catarina. This species is a small shrub that produces white flowers and berries with tens of small seeds (Baumgratz et al. 2025; Reginato 2016; Fig. 1). This species has unknown pollinators and seed dispersers, but most *Miconia* species are pollinated by vibrating bees and dispersed mainly by birds (Renner 1989; Messeder et al. 2021). After its first description in 1890, *M. ulei* has been recorded a few times, mostly in lowland areas with ombrophilous forest. Due to the few records, *M. ulei* is considered a rare species (Reginato 2016), currently assumed as under extinction risk (Bachman et al. 2024). However, these few records of *M. ulei* are concentrated in fragmented forest remnants in the AF domain. Given this scenario, the conservation status of *M. ulei* may be underestimated by ignoring the possible effects of forest fragmentation on population genetic parameters. Therefore, considering the ongoing environmental changes affecting the AF, genetic data are essential to evaluate the adaptive potential and support the long-term conservation of *M. ulei*.

Considering the importance of genetic conservation and the limited incorporation of genetic data into extinction risk assessments, we aimed to assess the conservation status of the rare species *Miconia ulei* by incorporating population genetic parameters. We investigated the genetic diversity, genetic structure, and effective population size of remnant populations in order to discuss possible conservation strategies. We hypothesized that habitat fragmentation affecting the rare species *M. ulei* has reduced genetic diversity and effective population size while increasing genetic differentiation among populations. We expected these patterns to be more pronounced than those reported for common species from the AF. Our findings can contribute to future conservation planning for *M. ulei* and improve the understanding of how habitat fragmentation affects rare species in the AF.

## Materials and methods

### Population sampling

**Fig. 2:**
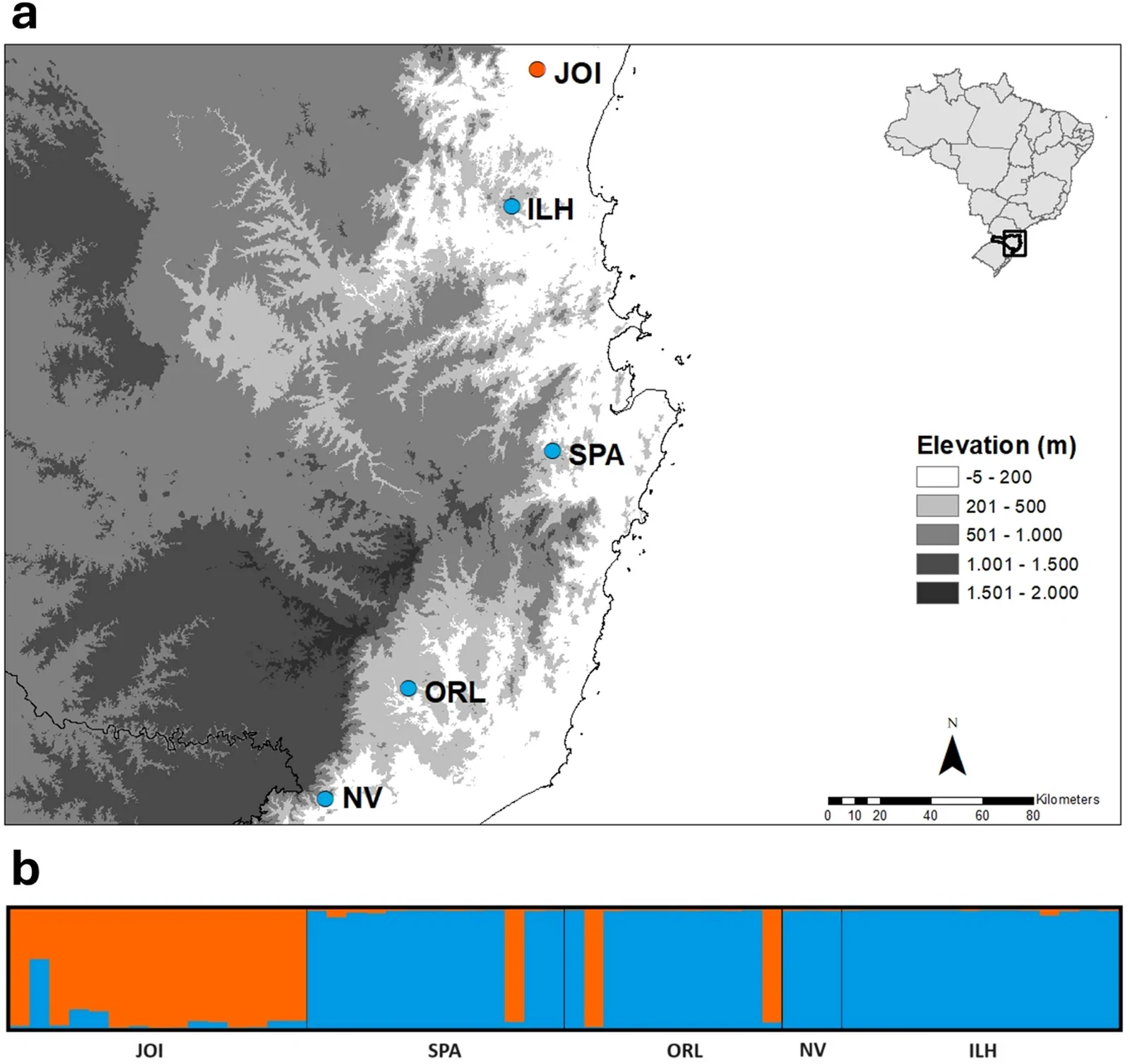
**a** Geographic distribution of sampled populations of *M. ulei* in Santa Catarina State, Brazil, labelled according to the population code (JOI = Joinville, ILH = Ilhota, SPA = São Pedro de Alcântara, ORL = Orleans, NV = Nova Veneza.). Populations are represented with colors of the main genetic clusters in the Structure analyses (see Results section). **b** Graphic representation of the genetic structure analysis conducted with STRUCTURE, with K = 2. Distinct colours represent distinct genetic clusters

We sampled 56 individuals of *M. ulei* from five populations distributed in the Atlantic Forest domain, ranging from 17 to 601 m above sea level (masl) (Table 1; Fig. 2a). We made additional efforts to locate three previously reported populations (*Santos et al. 303*; *Citadini-Zanette & Boff 2250*, *Korte & Kniess 3518*), but these were not found. Individuals were sampled at a minimum distance of 10 m to each other to reduce the probability of sampling ramets. Leaf tissue was collected from each individual and stored in zip-lock bags with silica gel until laboratory processing. For each population, one voucher was collected and deposited at herbarium FLOR (acronym following Thiers 2026).

**Table 1:** Sampled populations of *M. ulei*

Population codes	Locality - municipality	Altitude (m)	Sample size	Voucher	Lat (S)	Long (W)
JOI	Nova Brasília - Joinville	17 m	15	Goebel, G. et al. 338	26º22’24.1”	48º51’55.4”
ILH	Morro do Baú - Ilhota	226 m	14	Goebel, G. et al. 336	26º48’20.4”	48º57’10.5”
SPA	Caminho das Tropas - São Pedro de Alcântara	254 m	13	Goebel, G. et al. 335	27º34’17.9”	48º48’34.6”
ORL	Rio Novo - Orleans	268 m	11	Goebel, G. et al. 337	28º18’31.1”	49º19’02.0”
NV	Reserva São Francisco - Nova Veneza	601 m	3	Goebel, G. et al. 339	28º38’59.1”	49º36’31.7”

JOI = Joinville, ILH = Ilhota, SPA = São Pedro de Alcântara, ORL = Orleans, NV = Nova Veneza

### DNA extraction and sequencing

Genomic DNA extraction followed CTAB protocol (Doyle and Doyle 1987). After each extraction procedure, agarose gel electrophoresis was performed to evaluate the integrity of the genetic material. We used the Nanodrop spectrophotometer (Thermo Scientific) to verify the quality of the samples, and the Qubit fluorometer (Thermo Scientific) for DNA quantifications.

One sample (*Goebel et al. 335*) was selected for HTS (high-throughput sequencing). The genomic library was prepared using the SQK-LSK110 (Oxford Nanopore Technologies) Ligation Sequencing gDNA kit protocol, and sequencing was performed using the MinION Mk1B device (Oxford Nanopore Technologies), which uses an ion channel-type pore protein to sequence DNA (Clarke et al. 2009). The basecalling step was performed using the Guppy tool, version 6.5.7 + ca6d6af. The assembling of long reads into contigs was performed using the Flye software v2.9.2-b1786 (Kolmogorov et al. 2019). These steps were performed in the Laboratory of Developmental Physiology and Plant Genetics (LFDGV) of the Center for Agricultural Sciences at UFSC.

### SSR markers development, amplification and genotyping

The microsatellite (simple sequence repeat, SSR) loci prospection was performed using GMATA (Genome-wide Microsatellite Analyzing Tool Package) software (Wang and Wang 2016). The configuration was based on searching for a minimum of five repeat motifs in the case of di, tri and tetranucleotides, and a minimum of three repeat motifs for penta and hexanucleotides. SSRs sequences were selected from different contigs. Only nuclear regions were considered after searches using the BLAST platform (National Center for Biotechnology Information - NCBI) (https://www.ncbi.nlm.nih.gov/).

After initial prospection, the selected regions were validated in silico using virtual PCR (Polymerase Chain Reaction) and simulated gel electrophoresis available with the package SPCR (Simulated Polymerase Chain Reaction) (Cao et al. 2005). Afterwards, we selected 20 SSR regions for the second step validation by actual PCR in the thermocycler (Applied Biosystems), in order to optimize annealing temperature and DNA concentration.

After validation, we selected 12 SSR loci for genotyping (Table 2). The PCR technique used the M13 method, which consists of a fluorescent tail linked to the forward primer (Schuelke 2000). The PCR amplifications followed the final volume of 10 µL, containing: 1 µL of DNA, 6 µL of DreamTaq Master Mix 2x (Thermo Scientific), 1 µL of H2O, 0.5 µL of BSA, 0.5 forward primer, 0.5 µL of reverse primer, and 0.5 µL of M13 with HEX or FAM fluorescence. The thermocycler program was 94 °C for 5 min, followed by 30 cycles of 94 °C for 30 s, 72 °C for 45 s; followed by eight cycles of 94 °C for 30 s, 53 °C for 45 s, 72 °C for 45 s; with a final extension at 72 °C for 10 min, maintained at 4 °C. The PCR products were genotyped by capillary electrophoresis using an ABI 3500xL automated sequencer (Applied Biosystems) in multiplex reactions. Each reaction was performed with a final volume of 10 µL, following: 0.25 µL LIZ, 7.75 µL formamide, and 1 µL of each PCR product without dilution. The sizes of the microsatellite fragments were determined using GeneMapper version 3.2 (Applied Biosystems), according to established reading parameters, and the electropherograms were individually checked.

**Table 2:** Microsatellite (SSR) markers specifically developed for *M. ulei*. Ta = annealing temperature

Locus	Label	Primer’s sequence (5’ − 3’)	Motif	Allele size range (bp)	Ta (°C)
Mk 105	HEX	F: AGAGGCCCTTTTGTTGTCCT R: TGGAGTCGAATCGCAATTTT	(AT)8	399–403	60
Mk 1031	HEX	F: GACTGTCGGAAGGCAATCTC R: CGATCGGAACCACTTGATAA	(TA)6	124–145	63
Mk 1091	FAM	F: TCGGTAACAGCCAAAAGAGC R: CAATCCCCTCGTGAATGTTT	(TG)7	205–208	55
Mk 1117	HEX	F: TCATGATGGTTTGGATTGGA R: GAATACTGGCGAGGGGATATT	(TTA)5	386–418	55
Mk 1131	FAM	F: TTCCTTTTGCAAGCTCGTTT R: TGGTCATCATCAGGACTGGA	(TC)5	210–215	55
Mk 1245	HEX	F: CATCCAATCCGTTTGATCCT R: TCCGCCCTACAATCTCATTC	(GTG)6	378–379	55
Mk 1261	FAM	F: CAGCGGATCTTATTCGAAGG R: CAATGGAACTCCAAGCAGGT	(TC)7	300–301	59
Mk 1314	HEX	F: GCTCATATTTCGCCTTGTCC R: ATTCAACACAAAGCCCAACC	(AC)9	412–416	59
Mk 1417	FAM	F: AACATCCACGTCACATTTGC R: AGCACGGCTAGGATTGAGAA	(TTC)7	288–290	59
Mk 1422	FAM	F: TCCGCAATAATCCCCATAAA R: ATCTTCCGCTTGGAGACAAA	(AAC)5	226–240	55
Mk 1414	FAM	F: GCGCCTATGTCTTCGTTTTC R: AGTACGCCGTGGAAATTGTT	(GT)5	234–247	59
Mk 1327	HEX	F: CTTTCGGGCATCATCCTTAC R: GTTCCCTTGTCTTCCCCAAT	(CAT)6	377–384	59

### Inferring genetic diversity and structure

In order to evaluate null alleles or genotyping errors, we analysed genotyping data with the Micro-checker software (Van Osterhout et al. 2004). We estimated linkage disequilibrium (LD) presence and departures from the Hardy-Weinberg equilibrium (HWE) using the Genepop v.4.8.3 (Rousset 2008). In the LD analysis we considered a significance level of alpha = 0.05 and adjusted according to the Holm-Bonferroni correction for multiple comparisons (Holm 1979). We used the GenAIEx 6.5 software (Peakall and Smouse 2012) to estimate allele frequencies and genetic diversity indices, such as total number of alleles per locus (A), effective number of alleles per locus (Ae), observed heterozygosity (H_O_), unbiased expected heterozygosity (uH_E_), inbreeding coefficient (*F*_*IS*_) and the fixation index (*F*_*ST*_). To examine whether genetic divergence among populations is an effect of isolation by distance, we performed a Mantel test on GenePop Web (Rousset 2008) using the geographical and genetic distance (*F*_*ST*_/(1 − *F*_*ST*_) matrices.

To investigate the genetic structure, we used a Bayesian clustering algorithm in Structure software, version 2.3.4 (Pritchard et al. 2000). The simulations were run with *K* ranging from 1 to 5, considering 10 iterations for each *K* value. We used the admixture model, assuming independent allele frequencies, with a burn-in period of 100.000 iterations and a run length of 200.000 Markov chain Monte Carlo (MCMC) steps for each run. To determine the best number of genetic clusters, we implemented the Evanno test (Evanno 2005), analysing Δ*K* and MeanLnP(K), with the online tool Structure Selector (Li and Liu 2018). We performed an Analysis of Molecular Variance (AMOVA) on the F_ST_ values among and within populations by utilizing the Arlequin 3.5.2.2 software (Excoffier and Lischer 2010), testing the statistical significance with 10.000 permutations. The analysis considered the six loci without missing data. We also considered the pairwise *F*_*ST*_ to evaluate the genetic differentiation among populations.

### Inferring effective population size and bottlenecks

To estimate the effective population size (*N*_*e*_), we utilized the NeEstimator v.2.1 software (Do et al. 2014). We applied the linkage disequilibrium (LD) method, assuming random mating and isolated populations (Waples and Do 2010). In the LD method, singleton alleles were excluded and runs were performed without applying frequency restrictions. Confidence intervals were calculated using the Jackknife procedure (Jones et al. 2016). Although the populations analyzed comprised a small number of individuals, these estimates can still provide useful insights to support conservation decisions (Waples and Do 2010).

To investigate historical reductions in population size, we utilized the Bottleneck software version 1.2.02 (Piry et al. 1999). The analysis was based on three mutation models: Infinite Allele Model (IAM), Stepwise Mutation Model (SMM) and Two Phase Model (TPM). The SMM and TPM are considered the most appropriate models for microsatellites (Di Rienzo et al. 1994; Bhargava and Fuentes 2010). The statistical significance of the results was determined by the sign test and one-tail Wilcoxon test with 10.000 iterations (Luikart et al. 1998a). Mode-shift model was also evaluated in the Bottleneck software to investigate shifts in allele frequencies from the L-shaped distribution (Luikart et al. 1998b). Additionally, in Arlequin software, version 3.5, we performed the Garza-Williamson index to investigate evidence of population bottlenecks. We calculated M = k/r, where M = mean ratio of the number of alleles to the range in allele size; k = total number of alleles and r = the distance between alleles in number of repeats and the overall range in allele size (Garza and Williamson 2001).

### IUCN red list assessment

To assess the extinction risk of *M. ulei*, we utilized the IUCN Red List guidelines (IUCN 2024). We compiled and filtered all available records of *M. ulei* from online databases. We measured the geographic range through Extent of Occurrence (EOO) and Area of Occupancy (AOO) with the online tool Geospatial Conservation Assessment Tool (GeoCAT) (Bachman and Moat 2011) with default parameters. Information obtained through field sampling was used to confront IUCN criteria and categories. Population genetic data were also incorporated into the conservation assessment to provide complementary evidence regarding the species’ conservation status.

## Results

After HTS, we obtained 382.58 k reads and identified 2,865 microsatellite regions from the *M. ulei* genome. Dinucleotide motifs corresponded to 61%, while trinucleotides to 31.5%, and tetranucleotides to 3.4%. Among dinucleotides, most corresponded to AT/TA (27%), followed by CT/AG (11%).

All the 12 selected SSR *loci* were polimorphic. The Micro-checker analysis found no evidence of large allele dropout or stuttering. The presence of null alleles was suggested at three *loci* (1261, 1414, and 1245), based on homozygote excess detected in different populations. The genotypic linkage disequilibrium was not significant after the Holm-Bonferroni correction, indicating the independence between *loci*.

### Genetic diversity and structure

All the populations of *M. ulei* exhibited a low-moderate genetic diversity and low values of inbreeding coefficient (Table 3). The number of alleles per population ranged from 1.9 (NV) to 2.7 (ORL), and the effective number of alleles ranged from 1.7 (ILH) to 2.1 (JOI). Private alleles were found in JOI and ORL populations, with four and six alleles, respectively. The observed heterozigosity (H_O_) ranged from 0.389 (NV) to 0.481 (JOI), and unbiased expected heterozigosity (uH_E_) from 0.351 (ILH) to 0.489 (JOI). Inbreeding coefficient (*F*_*IS*_) ranged from − 0.297 (ILH) to 0.049 (ORL).

All populations presented significant deviations from HWE. Both heterozygote deficits and excesses were observed, and their occurrence varied among *loci* and populations. A deficit of heterozygotes was found in seven *loci*: 1031 (ORL), 1091 (SPA, NV, and ILH), 1261 (JOI and ORL), 1245 (JOI, SPA, ORL, and ILH), 1327 (JOI), 1417 (JOI and SPA), and 1422 (ORL). An excess of heterozygotes was found in seven *loci*: 105 (JOI, SPA, ORL, NV, and ILH), 1031 (SPA and ILH), 1117 (SPA, ORL, and ILH), 1131 (JOI, SPA, ORL, and ILH), 1314 (SPA and ILH), 1327 (SPA, ORL, and ILH), and 1414 (JOI).

**Table 3:** Genetic diversity indices for populations of *Miconia ulei*

Population	A (SE)	Ae (SE)	Ap	H_O_ (SE)	uH_E_ (SE)	F_IS_ (SE)
JOI	2.583 (0.260)	2.123 (0.208)	4	0.481 (0.104)	0.489 (0.059)	0.020 (0.184)
SPA	2.417 (0.288)	1.817 (0.193)	-	0.479 (0.141)	0.383 (0.076)	−0.090 (0.228)
ORL	2.750 (0.392)	1.911 (0.232)	6	0.452 (0.133)	0.402 (0.079)	0.049 (0.212)
NV	1.917 (0.288)	1.762 (0.241)	-	0.389 (0.141)	0.378 (0.100)	−0.165 (0.236)
ILH	2.000 (0.213)	1.690 (0.156)	-	0.470 (0.139)	0.351 (0.073)	−0.297 (0.229)

SE = Standard errors; A = mean number of alleles; Ae = mean effective number of alleles; Ap = number of private alleles; H_O_ = mean observed heterozigosity; uH_E_ = mean unbiased expected heterozigosity; F_IS_ = inbreeding coefficient

The STRUCTURE analysis had a best-fitting *K*-value of 2, indicating two genetic clusters within *M. ulei* (Fig. 2b). One of them was mostly composed of individuals from the JOI population, and the second cluster was composed of individuals from the other populations. Thus, a distinct genetic pattern is found in the JOI population, although some individuals from JOI show similarities with the other cluster. However, the Mantel test was not significant (*R* = 0.05; *p* = 0.3850), indicating no evidence of isolation by distance.

The AMOVA indicated that most of the genetic variation (96%) occurs within populations, while only a small fraction (4%) occurs among populations (F_ST_ = 0.044, *p* < 0.005).

The *F*_*ST*_ values ranged from 0.031 to 0.173 (see Supplementary Information 1, Table 4). The highest differentiation occurred between JOI and NV (0.162), and the lowest by ORL and SPA (0.031). In general, the JOI population exhibited a greater genetic differentiation in relation to the other populations of *M. ulei*.

### Effective population size and bottlenecks

The estimates of effective population size (*N*_*e*_) ranged from 1.2 (95% CI = 0.6–2.5) individuals in the JOI population to 21.1 (95% CI = 1.1 - Infinite) in the SPA population (see Supplementary Information 1, Table 5). Infinite *Ne* values were obtained in NV and ILH populations, indicating insufficient sampling for reliable estimates. The NV population was represented by only three individuals, despite intensive searches in the area.

The Bottleneck test indicated evidence of bottleneck events in the JOI, NV, and ILH populations of *M. ulei* (see Supplementary Information 1, Table 5). The analysis of heterozygosity excess indicated that under the IAM mutation model only ILH presented statistical support for a bottleneck event. The statistical significance was obtained in JOI, NV, and ILH populations under SMM and TPM mutation models, indicating a positive signal for a bottleneck event. In the case of the distribution of allele frequencies, JOI, NV and ILH presented a shifted frequency distribution curve, and in the M-ratio model only NV and ILH presented a bottleneck pattern.

### IUCN red list assessment

The data obtained from *M. ulei* populations were compared to all criteria and categories of the IUCN Red List guidelines. We estimated EOO as 8,678 km² and AOO as 52 km². Three populations were not found during field efforts and were assumed to be locally extinct. These populations were therefore not included in the EOO and AOO calculations. Indeed, we verified that the most severe threats which *M. ulei* populations are facing are agriculture, forestry expansion, urbanisation, and the presence of exotic species within their habitats (see Supplementary Information 2, Fig. 3). Considering the restricted AOO, the apparent fragmentation of populations, and the continuing decline in habitat quality, *M. ulei* is assessed here as Endangered (EN), following criteria B2a, b(iii). The complete assessment is in preparation to be submitted to the IUCN Red List (https://www.iucnredlist.org/*).*

## Discussion

In this study, we investigated the genetic diversity, population genetic structure, and effective population size of remnant populations of *Miconia ulei*, a rare plant species from the Southern Brazilian Atlantic Forest (AF), in order to discuss possible conservation actions. For this aim, we developed a specific set of microsatellite markers and investigated these genetic parameters in five *M. ulei* populations that are in secondary forest remnants. We found that *M. ulei* populations exhibited low to moderate genetic diversity, overall low genetic structure, and signs of past bottleneck events. Our findings directly contribute to the assessment of the conservation status of this rare species and have implications for its conservation strategies.

### Genetic diversity and structure

The populations of *M. ulei* showed overall low to moderate genetic diversity, with low allelic diversity across loci (1.9 to 2.7 alleles per locus) and moderate heterozygosities (H_O_: 0.389–0.481; uH_E_ 0.351–0.489). Our findings contrast with patterns found for common plant species in the AF, with overall greater genetic diversity (Melo and Franceschinelli 2016; Pereira et al. 2022; Beise et al. 2022; Godoy et al. 2023). For instance, in populations of *Cabralea canjerana* (Meliaceae), an endemic and threatened species tree from the AF, the number of alleles found varied from 6 to 10 per locus, with average H_E_ of 0.7 and H_O_ of 0.7 (Melo and Franceschinelli 2016). Our findings are consistent with the expected pattern for endemic woody species, which tend to exhibit lower genetic diversity than broadly distributed species (Hamrick et al. 1992; Hamrick and Godt 1996). Despite that, our results on the genetic diversity of *M. ulei* also reflect the long history of deforestation in the AF. This process often causes the loss of rare alleles, a pattern that may not be immediately detected by heterozygosity-based metrics (Nei et al. 1975; Cornuet and Luikart 1996), but is evident in the reduced allelic diversity, as seen in the case of *M. ulei*. Thus, our findings suggest that anthropogenic deforestation may have had a stronger negative effect on genetic diversity in rare plant species than in common species in the AF. However, this interpretation should be considered with caution, as differences between rare and common species may also reflect variation in geographic distribution, reproductive biology, demographic history, and phylogenetic relatedness, rather than rarity alone.

Overall, *M. ulei* showed low genetic differentiation among populations, although this pattern was not uniform across population pairs. The STRUCTURE analyses revealed only two genetic groups within *M. ulei*: (1) the JOI population and (2) the SPA, ILH, ORL, and NV populations, with some degree of admixture. The low overall differentiation was largely driven by the weak differentiation among SPA, ILH, ORL, and NV, whereas JOI was clearly differentiated from the other populations. Contrary to our expectations of strong population genetic structure due to gene flow disruption caused by habitat fragmentation, the observed pattern suggests that most populations remain genetically similar, with JOI accounting for much of the observed differentiation (Aguilar et al. 2008; Frankham et al. 2019b). Moreover, our findings contrast with the patterns of population genetic structure commonly reported for common plant species in the AF. Studies involving a similar number of sampled populations have generally identified a greater number of genetic clusters, accompanied by higher levels of admixture among gene pools (Santos et al. 2019; Carvalho et al. 2020). For example, in *Eschweilera ovata* (Lecythidaceae), a common tree species from the northeastern AF, analyses of five populations revealed three genetic clusters, with extensive gene pool sharing among individuals assigned to different clusters (Santos et al. 2019). Because isolation by distance was not supported by the Mantel test, the observed population genetic structure of *M. ulei* may be influenced by its reproductive system rather than by geographic distance alone.

Indeed, most genetic variation of *M. ulei* occurs within populations, suggesting a strict outcrossing reproductive system. In general, low genetic differentiation among plant populations is associated with strictly outcrossing reproductive systems (Hamrick and Loveless 1989). Despite the lack of specific studies, *M. ulei* likely has a strictly outcrossing reproductive system that relies on buzzing bees for pollination, like most melastomes (Renner 1989). Supporting this perspective, two populations, JOI and ORL, exhibited F_IS_ values close to zero, indicating limited inbreeding within populations. In addition, the JOI population occurs at sea level, whereas all other populations are located above 200 m elevation. This elevational variation can influence the activity and availability of bee pollinators, affecting gene flow among populations (Young et al. 1996). For instance, in the bee-pollinated *Tibouchina pulchra* (Melastomataceae), population genetic structure is associated with the elevational gradient in the AF (Brito et al. 2016). In addition, the distinct genetic position of JOI may also be related to its geographic location in the lowland portion of the municipality of Joinville, with the Serra do Mar potentially contributing to reduced gene flow between JOI and more inland populations. Thus, the strictly outcrossing reproductive system, together with environmental and geographic heterogeneity across the species’ distribution, may have contributed to the population genetic structure of *M. ulei*.

We also detected a relatively strong negative F_IS_ value for the ILH population compared with the other populations of *M. ulei*. This pattern may reflect several non-mutually exclusive processes. For instance, the negative F_IS_ may be potentially associated with non-random mating among genetically dissimilar individuals, the presence of a small effective population size not detected by our analyses, or natural selection favoring heterozygous individuals (Allendorf et al. 2013). However, given the small sample size of the ILH population and the relatively high standard error associated with the F_IS_ estimate (− 0.297 ± 0.229), this result should be interpreted with caution. Therefore, we cannot rule out the possibility that the observed negative F_IS_ reflects sampling variation rather than a biologically meaningful process.

According to our expectations, the sampled populations of *M. ulei* exhibited low effective sizes (*N*_*e*_ = 1.2–21), and some populations showed signals of past bottleneck events. The Bottleneck analysis provided evidence of historical population size reductions in JOI and ILH, whereas the signal detected for NV should be interpreted with caution because of its small sample size. Although intensive field searches were conducted in the area, no additional individuals were found, highlighting the rarity of this population. Therefore, the *Ne* estimate and bottleneck results for NV should be interpreted cautiously. For JOI and ILH, however, the evidence of bottleneck events is consistent with the low genetic diversity observed in *M. ulei*. Bottleneck events are documented for common and rare species in the AF (Conson et al. 2013; Santos et al. 2019; Augustin et al. 2025). In *Luehea divaricata* (Malvaceae), a common species from AF, this pattern was evidenced in all the nine analyzed populations (Conson et al. 2013). These results are in line with the long history of forest exploitation in southern Brazil, which has been ongoing over the last 300 years (Carola 2010; Carlucci et al. 2021). Given the absence of major climate-driven vegetation contractions in the region during at least the last millennium and considering that the AF reached its maximum expansion during the Holocene (Behling 1995; Behling and Negrelle 2001), the observed genetic patterns are more plausibly attributed to recent anthropogenic impacts rather than to natural climatic fluctuations. The habitat fragmentation is related to reductions in population sizes and in the effective population sizes (Aguilar et al. 2008; Frankham et al. 2019b), and bottleneck events are a well-documented consequence of this process (Young et al. 1996; Aguilar et al. 2008). Thus, the demographic signals detected in *M. ulei* may reflect the consequences of historical habitat loss and fragmentation in the AF, which may have reduced both population size and genetic diversity over time.

This study presented some limitations, particularly the small population sizes sampled, which may influence certain genetic estimates but also reflect the current demographic condition of *M. ulei*. Nevertheless, the results provide important insights into the conservation of the species, revealing signs of genetic loss, identifying ongoing threats to its habitat, and allowing us to propose conservation actions. In addition, this study provides valuable genetic data for a rare Atlantic Forest species, allowing comparisons with more common and widespread species and contributing to a broader understanding of how habitat fragmentation affects species with different ecological and demographic characteristics.

### Conservation implications

Our results highlight the need for conservation measures for *M. ulei*, a rare plant species in the AF domain. According to our analyses based on IUCN Red List criteria, the species may be categorized as Endangered (EN). Although the IUCN criteria do not explicitly incorporate genetic data, our results provide important complementary evidence regarding the conservation and demographic status of the species, including signatures of population reduction detected in some populations. We also found reduced genetic variability and low effective population sizes, suggesting that only a small number of individuals are contributing genetically to subsequent generations. *N*_*e*_s lower than 500 could potentially reduce population’s ability, and it is a relevant factor to be considered in extinction risk assessments (Jamieson and Allendorf 2012; Hoban et al. 2020). Therefore, our findings demonstrate that genetic data can substantially improve the assessment of the conservation status of *M. ulei*.

First, we suggest including *M. ulei* in the IUCN Red List, as well as in national and regional lists of threatened flora. These actions will enable legal recognition and access to funding for research and conservation planning. A complete assessment of conservation status is currently in preparation for submission to the IUCN Red List. At the regional level, we recommend its inclusion in the Red List of Santa Catarina, the latest version published in 2014 (CONSEMA 2014).

Second, we suggest in situ conservation strategies for the long-term persistence of *M. ulei*. Field observations revealed ongoing habitat degradation associated with exotic species, agricultural expansion, urbanisation, and continued forest loss in Santa Catarina state (MapBiomas 2024). Therefore, habitat protection, removal of local threats, and long-term population monitoring are essential conservation actions, especially because most populations occur on private lands (JOI, SPA, ORL, and NV), reinforcing the importance of engaging local communities and landowners. Although the ILH population is located within a protected area, its low genetic diversity suggests that legal protection alone has not been sufficient to reverse the historical effects of habitat degradation. In this context, the enforcement of Brazilian environmental legislation is particularly important, since *M. ulei* is associated with riparian areas that should legally maintain native vegetation cover (Brazil 2012), although many remain degraded or unprotected. Habitat restoration in these areas may increase population size and expand suitable habitat. However, because the natural recovery of rare species may be limited in highly degraded areas lacking propagule sources, active reintroduction should also be considered within restoration programs following conservation translocation guidelines (Godefroid et al. 2011; Kirkwood 2013).

Third, we also suggest that *ex situ* conservation strategies are necessary for *M. ulei*. Genetic rescue may represent an important strategy to mitigate the loss of genetic diversity in *M. ulei* by introducing new alleles into populations with low genetic diversity (Liddell et al. 2021; Ralls et al. 2018; Frankham et al. 2019b). The relatively low population differentiation and absence of strong inbreeding signals support the feasibility of this approach. Our results suggest that reintroduction programs should prioritize maximizing the number of introduced individuals while carefully selecting source populations and conducting long-term demographic and genetic monitoring (Hoffmann et al. 2021; Van Rossum and Hardy 2022). In this context, seedling production represents a fundamental step for both reintroduction and genetic rescue programs. In a complementary study, *M. ulei* showed high seed viability and germination rates (85%), with no clear evidence of inbreeding depression, and seeds remained viable for at least seven months under low-temperature storage conditions (Goebel et al. *in press*.). These characteristics support the feasibility of *ex situ* conservation strategies, including seedling production, cultivation in nurseries and botanical gardens, and future seed banking initiatives.

In the long-term, further research on the species’ biology, including its reproductive system, phenology, and seedling establishment, is important to support conservation strategies. Additionally, studies on seed longevity and tolerance to negative temperatures, as used in cryopreservation practices, would be valuable for seed bank implementation. The inclusion of *M. ulei* in broader conservation initiatives is also recommended. In this context, the *PAT Planalto Sul*, coordinated by the Instituto do Meio Ambiente de Santa Catarina (IMA), represents a relevant framework, as it promotes the conservation of threatened species through a territorial and integrative approach. Specifically, *M. ulei* fits actions focused on habitat protection and recovery, invasive species management, reduction of native vegetation loss, reintroduction in suitable habitats, and the identification and monitoring of priority areas and populations. Therefore, given its conservation status and ecological context, *M. ulei* should be considered a priority species for inclusion in such programs.

## Conclusions

This study is the first population genetic assessment of *Miconia ulei*, a rare species from the Southern Atlantic Forest, providing important insights into its conservation status and genetic vulnerability. Our findings indicate that the species can be classified as at risk of extinction, with evidence of loss of genetic diversity, low effective population sizes, and evidence of past bottleneck events, which we attribute to habitat fragmentation and anthropogenic disturbance in the Atlantic Forest. Thus, conservation actions should be implemented soon, both in the short- and long-term. The first step should be its inclusion in official red lists to gain visibility and legal support. Increasing population sizes and promoting gene flow are essential measures to promote the persistence of *M. ulei* and can be achieved in different ways: guaranteeing an appropriate habitat, and removing its threats, while allowing the entry of more individuals into its populations. There is potential to seedling production, although more studies should be done to evaluate seedling establishment. Future actions should encompass more information about biological and ecological aspects of the species, monitoring its populations, and guaranteeing an appropriate habitat for species maintenance. Overall, our findings demonstrate the importance of incorporating genetic data into conservation assessments of rare species and contribute to a broader understanding of how habitat fragmentation affects rare plant species in the Atlantic Forest.

## Supplementary Information

Below is the link to the electronic supplementary material.

Supplementary material 1 (DOCX 459.0 kb)

Supplementary material 2 (DOCX 23.1 kb)

## Data Availability

No datasets were generated or analysed during the current study.
