# Supplementary material for: Genetic diversity and conservation of a rare Miconia species (Melastomataceae) from the Brazilian Atlantic Forest
Source: Genetica. 2026 Oct 1;154(1):45. doi: 10.1007/s10709-026-00296-1 (PMC13630976; doi:10.1007/s10709-026-00296-1)
Supplement: Supplementary file 1 [file 10709_2026_296_MOESM1_ESM.docx]

**SUPPLEMENTARY INFORMATION**

**Genetic diversity and conservation of a rare *Miconia* species (Melastomataceae) from the Brazilian Atlantic Forest**

Gabriela Goebel¹ *, Ana Flávia Augustin ² ⁵, Eduardo Koerich Nery ³, Yohan Fritsche ² ⁶, Valdir Marcos Stefenon ² ⁷, Duane Fernandes Lima ⁴, Mayara Krasinski Caddah¹ ⁸

¹Programa de Pós-graduação em Biologia de Fungos, Algas e Plantas, Universidade Federal de Santa Catarina, Centro de Ciências Biológicas, Departamento de Botânica, R. Eng. Agronômico Andrei Cristian Ferreira, 216, 88040-535 Florianópolis, SC, Brasil.

²Programa de Pós-graduação em Recursos Genéticos Vegetais, Universidade Federal de Santa Catarina, Centro de Ciências Agrárias, Departamento de Fitotecnia, Rod. Admar Gonzaga, 1346, 88034-000 Florianópolis, SC, Brasil.

³Universidade Federal do ABC, Centro de Ciências Naturais e Humanas, Alameda da Universidade, 09606-045 São Bernardo do Campo, SP, Brasil  [eduardo.k.nery@gmail.com](mailto:eduardo.k.nery@gmail.com) / https://orcid.org/0000-0003-4636-1285

⁴Universidade Estadual do Centro-Oeste, Setor de Ciências Agrárias e Ambientais, Departamento de Ciências Biológicas, Alameda Élio Dalla Vecchia, 838, 85040-167 Guarapuava, PR, Brasil duaneflima@gmail.com / https://orcid.org/0000-0003-4240-9077

⁵ afaugustin@gmail.com / https://orcid.org/0000-0001-6794-0513

⁶ y.fritsche@ufsc.br / https://orcid.org/0000-0002-1048-071X

⁷ valdir.stefenon@ufsc.br /  <https://orcid.org/0000-0003-1091-700X>

⁸ mayaracaddah@gmail.com / https://orcid.org/0000-0003-2285-208X

*Corresponding author: Gabriela Goebel / [gabriela.goebel6@gmail.com](mailto:gabriela.goebel6@gmail.com)

**Fig. 3** Records of historical and current occurrence sites of *Miconia ulei*. (a, b) Urban expansion; (c–f) agricultural expansion; (g) forestry expansion; and (h, i) occurrence of the exotic species *Pilea cadieri* and *Hedychium coronarium*, respectively, within *M. ulei* habitat. Photos by G. Goebel (a-c, e-i) and from Google Earth (d).
