# Supplementary material for: Genetic diversity and conservation of a rare Miconia species (Melastomataceae) from the Brazilian Atlantic Forest
Source: Genetica. 2026 Oct 1;154(1):45. doi: 10.1007/s10709-026-00296-1 (PMC13630976; doi:10.1007/s10709-026-00296-1)
Supplement: Supplementary file 2 [file 10709_2026_296_MOESM2_ESM.docx]

**SUPPLEMENTARY INFORMATION**

**Genetic diversity and conservation of a rare *Miconia* species (Melastomataceae) from the Brazilian Atlantic Forest**

Gabriela Goebel¹ *, Ana Flávia Augustin ² ⁵, Eduardo Koerich Nery ³, Yohan Fritsche ² ⁶, Valdir Marcos Stefenon ² ⁷, Duane Fernandes Lima ⁴, Mayara Krasinski Caddah¹ ⁸

¹Programa de Pós-graduação em Biologia de Fungos, Algas e Plantas, Universidade Federal de Santa Catarina, Centro de Ciências Biológicas, Departamento de Botânica, R. Eng. Agronômico Andrei Cristian Ferreira, 216, 88040-535 Florianópolis, SC, Brasil.

²Programa de Pós-graduação em Recursos Genéticos Vegetais, Universidade Federal de Santa Catarina, Centro de Ciências Agrárias, Departamento de Fitotecnia, Rod. Admar Gonzaga, 1346, 88034-000 Florianópolis, SC, Brasil.

³Universidade Federal do ABC, Centro de Ciências Naturais e Humanas, Alameda da Universidade, 09606-045 São Bernardo do Campo, SP, Brasil / eduardo.k.nery@gmail.com / https://orcid.org/0000-0003-4636-1285

⁴Universidade Estadual do Centro-Oeste, Setor de Ciências Agrárias e Ambientais, Departamento de Ciências Biológicas, Alameda Élio Dalla Vecchia, 838, 85040-167 Guarapuava, PR, Brasil / duaneflima@gmail.com / https://orcid.org/0000-0003-4240-9077

⁵ afaugustin@gmail.com / https://orcid.org/0000-0001-6794-0513

⁶ y.fritsche@ufsc.br / https://orcid.org/0000-0002-1048-071X

⁷ valdir.stefenon@ufsc.br / https://orcid.org/0000-0003-1091-700X

⁸ mayaracaddah@gmail.com / https://orcid.org/0000-0003-2285-208X

*Corresponding author: Gabriela Goebel / gabriela.goebel6@gmail.com

**Table 4**: Pairwise *F_ST_* values among populations of *Miconia ulei* sampled in this study*.*

| **Populations** | **JOI** | **SPA** | **ORL** | **NV** | **ILH** |
| --- | --- | --- | --- | --- | --- |
| JOI | 0.000 |  |  |  |  |
| SPA | 0.148 | 0.000 |  |  |  |
| ORL | 0.142 | 0.031 | 0.000 |  |  |
| NV | 0.173 | 0.065 | 0.063 | 0.000 |  |
| ILH | 0.162 | 0.037 | 0.038 | 0.040 | 0.000 |

**Table 5:** Bottleneck analysis under the heterozygosity excess, allele frequency distribution, and M-ratio methods. The values highlighted in bold have statistical support for a genetic bottleneck signature. He: *Loci* with heterozygosity excess; p*: Statistical significance of the Wilcoxon test for multilocus analysis of heterozygosity excess; Afd: Allele frequency distribution. *Ne* = effective population size; CI = confidence intervals.

|  |  | **IAM** |  | **SMM** | | **TPM** | |  |  |
| --- | --- | --- | --- | --- | --- | --- | --- | --- | --- |
| **Pop** | ***N_e_***  (95% CI) | *He* | p* | *He* | p* | *He* | p* | **Afd** | ***M-ratio*** |
| JOI | 1.2 (0.6 - 2.5) | 10 (p=0.06) | **0.001** | 10 (**p=0.01**) | **0.02** | 10 (**p=0.01**) | **0.001** | **Shifted** | 0.768 |
| SPA | 21.1 (1.1- Infinite) | 8 (p=0.06) | **0.016** | 6 (p=0.48) | 0.278 | 8 (p=0.08) | 0.096 | L-shaped | 0.685 |
| ORL | 1.2 (0.2 - 21.5) | 7 (p=0.22) | 0.161 | 5 (p=0.41) | 0.687 | 5 (p=047) | 0.347 | L-shaped | 0.772 |
| NV | Infinite (0.2 - Infinite) | 7 (p=0.07) | **0.004** | 7 (**p=0.03**) | **0.004** | 7 (**p=0.02**) | **0.004** | **Shifted** | **0.550** |
| ILH | Infinite (46.9 - Infinite) | 8 (**p=0.02**) | **0.007** | 8 (**p=0.03**) | **0.014** | 8 (**p=0.02**) | **0.010** | **Shifted** | **0.603** |
